# Predictors of the surgical outcome of propeller perforator flap reconstruction, focusing on the effective safe distance between the perforator and the wound edge

**DOI:** 10.1186/s12891-021-04522-z

**Published:** 2021-07-29

**Authors:** Peng Wang, Fang Lin, Yunhong Ma, Jianbing Wang, Ming Zhou, Yongjun Rui

**Affiliations:** Department of Orthopaedic Surgery, Wuxi No. 9 People’s Hospital Affiliated to Soochow University, No. 999 Liangxi Road, Wuxi, 214000 China

**Keywords:** Perforator flap, Propeller flap, Lower limb, Risk factors

## Abstract

**Background:**

Soft tissue defects in the distal third of the leg and malleolus are difficult to cover and often require free tissue transfer, even for small-sized defects. Propeller flaps were designed as an alternative to free tissue transfer, but are reportedly associated with high complication rates. The aim of our study was to assess our institutional experience with the propeller flap technique and to predict its outcome in lower-limb reconstruction.

**Methods:**

All patients who had undergone propeller flap reconstruction of a distal leg defect between 2013 and 2018 were included. Demographic, clinical, and follow-up data were analyzed.

**Results:**

Complications occurred in 17 of 82 propeller flaps (20.7%), comprising 11 cases of partial necrosis and six of total necrosis. There were no significant differences in age, sex, body mass index smoking, diabetes mellitus, and soft tissue defect sites between the groups of patients with versus without flap necrosis (*p* > 0.05). In univariate analysis, there were also no significant differences between these two groups in the length and width of the fascial pedicle, and the ratio of the flap length to the flap width (*p* > 0.05). Interestingly, there were significant differences between the two groups in the distance between the flap perforator, the shortest distance from the perforator to the defect location, and the rotation angle of the flap (*p* < 0.05). In multivariable logistic regression analysis with odds ratios (ORs) and 95% confidence intervals (95% CIs), the shortest distance from the perforator to the defect location was a significant risk factor for flap complications (*p* = 0.000; OR = 0.806). Receiver operating characteristic curve analysis showed that when the shortest distance from the flap to the wound was less than 3.5 cm, the necrosis rate of the flap was markedly increased (AUC = 76.1); this suggests that the effective safe flap–wound distance was 3.5 cm.

**Conclusions:**

Propeller flaps are a reliable option for reconstruction in carefully selected patients with traumatic defects of the lower limb and malleolus. We found that the effective safe distance was 3.5 cm from the flap to the wound.

## Background

The middle and lower limb and ankle contain a limited amount of soft tissue and have poor skin elasticity. Traumatic injuries to these regions often result in the exposure of bones and tendons, and it is difficult to directly close these defects, even for small defects. Therefore, flap covering is often required. In traumatic cases, the soft tissue coverage in this area is often further reduced due to extensive soft tissue destruction and the multiple incisions required for complex internal fixation. Moreover, the anatomical region of the internal fixation has limited soft tissue relaxation, which further increases the demand for soft tissue coverage. These factors often limit the use of traditional local flaps.

The main advantages of the propeller flap for the treatment of calf defects include a short operation time, shortened hospital stay, reduced patient costs, and location of the flap sites adjacent to the defect sites [1–3]. The reported complication rates associated with propeller flaps range widely from 8.3 to 42% [3–11], and the main complications are venous congestion and partial flap necrosis. Partial necrosis of skin flaps is affected by patient factors (sex, age, defect location), flap factors (rotation point position, flap proximal position, flap length and width, fascial pedicle length and width, total flap length, aspect ratio of the flap), and operator factors. The necrosis rate of skin flaps is reportedly increased in elderly patients with diabetes mellitus and peripheral vascular disease [12, 13]. Kelahmetoglu et al. [14] described a surgical modification that allows propeller perforator flaps to cover pressure sores at various locations; they used the propeller perforator flap concept based on the detection of newly formed perforator vessels located 1 cm from the wound margin and stimulated by the chronic inflammation process. To reduce the flap necrosis rate, the flap perforator should not be too close to the wound surface or located too far from the wound. However, no study has quantitatively analyzed the closest safe distance between the perforator and the wound, that is, the effective safe distance.

In this study, we focused on the lower limb because partial necrosis is more frequent for PPF (Perforator Propeller Flap) located on legs than in other locations. We retrospectively analyzed the records of patients who underwent propeller flap treatment in our hospital to assess the distance from the perforator to the propeller flap. The aim of the study was to determine the shortest safe distance from the perforator to the wound to increase the success rate and reduce the incidence of flap necrosis.

## Methods

All adult patients who received propeller flap treatment from 2013 to 2018 were included in this single-center study. The study population comprised 82 patients (47 men and 35 women) with an average age of 36.5 years (range 18–65 years). All patients had soft tissue defects in the lower legs that required flap reconstruction. The indications for propeller flaps instead of free flaps included traumatic defects in the distal third of the leg, the need for flaps to cover an area less than 5 cm in diameter, perforator arteries that were detectable by Doppler, and unsuitability for complex microvascular surgery. The exclusion criteria were: age less than 18 years or more than 65 years; local peripheral vascular disease, chronic lymphedema, or deep vein thrombosis; other pathological defects; venous, neuropathic, and malignant ulcers; Gustilo type IIIC injuries. Patients with any of these exclusion criteria underwent free flap reconstruction rather than propeller flap reconstruction. Table 1 lists the demographic data of the patients. All operations were performed by a single surgeon.

**Table 1:** Patient demographic data

Characteristics	Value
No. of patients	82
Mean age (range)	36.5(18–65)
Mean body mass index, kg/m	22.5
Diabetes mellitus, no. (%)	12 (14.6)
Smoking, no. (%)	34 (41.5)
Previous radiotherapy, no. (%)	1 (1.2)
Defect location, no	
Medial malleolus	31
External malleolus	8
Lower tibia	32
Middle tibia	11
Cause of the defect, no	
Infection	20
Trauma	62
Gustilo-Anderson type (%)	
II	6 (7.3)
IIIA	19 (23.2)
IIIB	57 (69.5)
Defect size (cm^2^)	12.4
Source vessel	
anterior tibial artery	12
posterior tibial artery	62
peroneal artery	8
Complications	
Vascular crisis. no.(%)	21 (25.6)
artery	2
vein	19
Infect. no.(%)	11 (13.4)
Superficial infection	10
Deep infection	1
Flaps necrosis. no. (%)	17 (20.7)
Partial necrosis	11
total necrosis	6
Aesthetic outcome	
5	2
4	35
3	37
2	5
1	3

The criteria used to define survival of the skin flap were: the skin flap was fully alive, and the wound was healed at the first stage. Partial flap necrosis was defined as necrosis of less than 50% of the total flap area. The flap length was defined as the maximum length of the longitudinal axis of the flap; the flap width was defined as the maximum width of the flap; the total flap length was defined as the length of the flap plus the length of the fascia; the length–width ratio was defined as the ratio of the total flap length to the width of the fascial pedicle. Table 2 lists the factors indicative of flap success and failure. In receiver operating characteristic (ROC) curve analysis for the optimal cutoff value of the arc of rotation, maximal statistical significance was achieved at an arc of rotation threshold of 135°. Therefore, the patients were stratified into those with an arc of rotation of less than 150° and those with an arc of rotation of 150–180° for further comparative analysis [15]. The flap length was equal to the sum of the large oar and the small oar, and the length of the small oar was measured as the distance from the fulcrum to the wound surface.

**Table 2:** Univariate analysis of the risk factors for vertebral compression fractures

Characteristics	Flaps necrosis groups		*p* Value
	Yes	No	
No. of flaps	17	65	
Mean age, y	45.9 ± 9.3	41.0 ± 10.8	0.094^a^
Mean body mass index, kg/m2	22.7 ± 3.1	22.3 ± 3.0	0.619^a^
Sex			0.873^b^
Male	8	32	
Female	9	33	
Location			0.676^b^
Middle tibia	5	15	
Lower tibia	7	26	
Lateral malleolus	3	8	
Medial malleolus	2	16	
Arc of rotation			0.010^b^
< 150	4	38	
150–180	13	27	
Diabetes mellitus			0.693^b^
Yes	3	9	
No	14	56	
Smoking			0.562^b^
Yes	6	28	
No	11	37	
Distance from perforator to center of defect	8.4 ± 2.3	9.4 ± 2.6	0.151^c^
Nearest distance from perforator to defect location	3.0 ± 0.9	3.8 ± 1.0	0.005^c^
Time from injury to definitive surgical procedure (days)	11.2 ± 4.2	10.0 ± 3.5	0.220^c^
Operative time (min)	160.3 ± 23.7	166.5 ± 18.7	0.256^c^
Length(cm)	12.4 ± 3.2	11.8 ± 3.7	0.573^c^
Width(cm)	5.6 ± 2.1	6.2 ± 1.4	0.428^c^
Flap Size(cm)	62.5 ± 14.6	58.9 ± 13.5	0.317^c^
length–width ratio	2.9 ± 0.9	2.7 ± 1.1	0.182^c^

^a^ independent t-test; ^b^ Chi-square tests; ^c^Mann-Whitney U tests * *P* < 0.05

### Surgical steps

#### Preoperative positioning

Preoperative planning was done using computed tomographic angiography (CTA) and/or color Doppler ultrasound to locate the perforating vessels.

#### Skin flap design

**Fig. 1:**
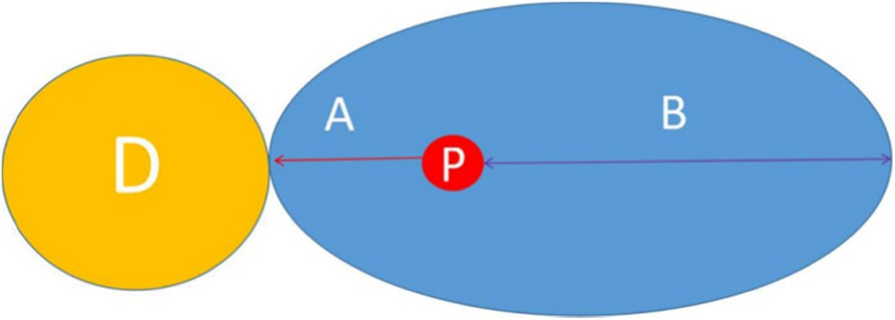
Drawing depicting a propeller flap. The distance of A (shortest distance from the perforator to the defect) in a propeller flap. A flap rotation of 180° is possible if there is available tissue in the linear axis (A) connecting the defect (D) (yellow area) and the emerging point of the perforator vessel (P) (red circle) on the other side of the perforator, compared with the position of the defect (B) (blue area)

**Fig. 2:**
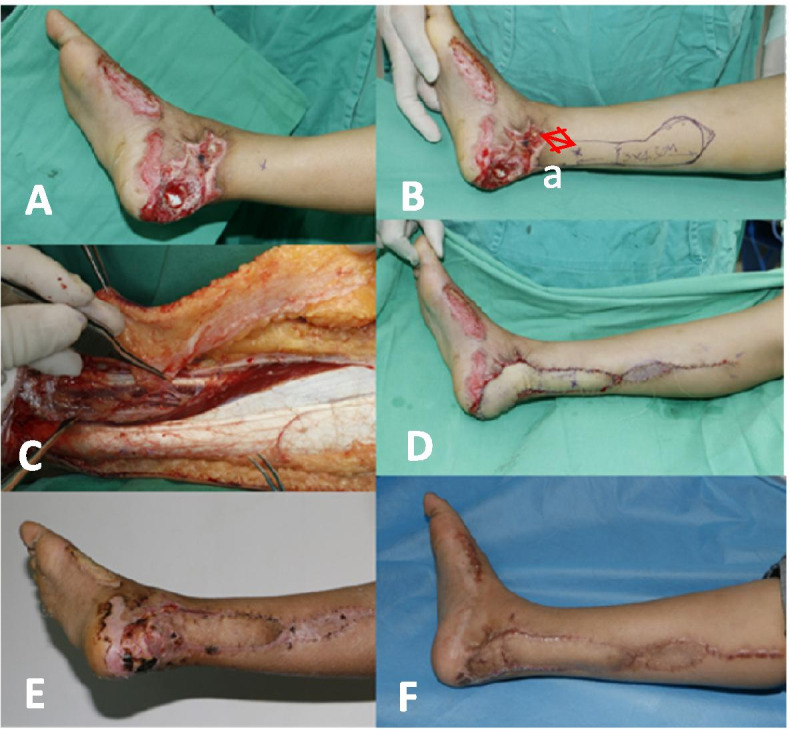
Photographs illustrating the propeller flap operative technique. **A**, **B** Case example showing typical locations and marking of perforators from the posterior tibial arteries. **C** The incision along one margin of the proposed flap. **D** Final defect coverage with the propeller flap and primary closure of the donor site. **E** Partial flap necrosis at 3 weeks postoperatively. **F** Healing and excellent contour of the reconstructed ankle at 6 months postoperatively

**Fig. 3:**
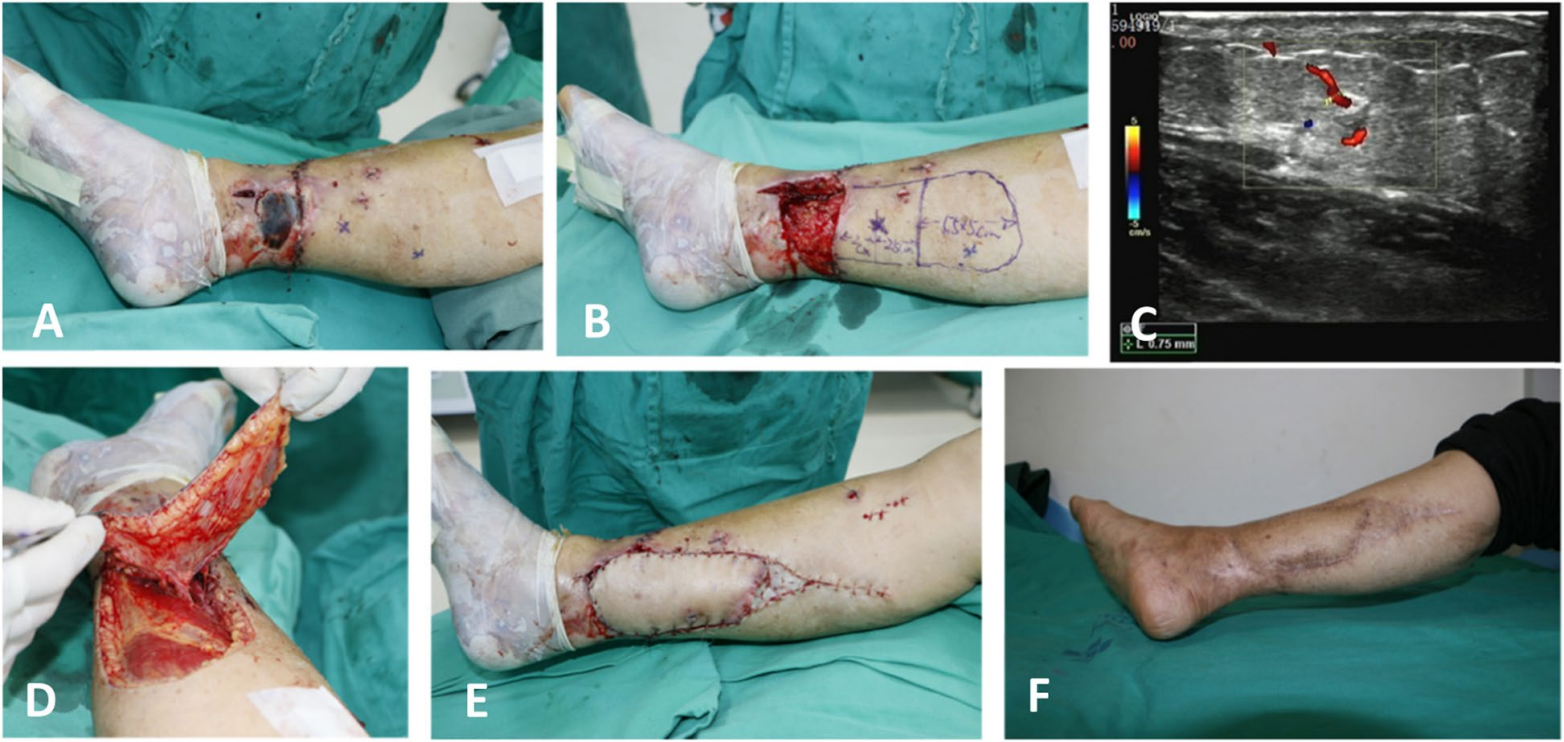
Photographs illustrating the propeller flap operative technique. **A**, **B** Photographs showing the skin defect overlying the fracture and the markings for the posterior tibial artery propeller flap. **C** Tracing of the perforator using a Doppler device. **D** Color of the flap before the 180-degree rotation. **E** Final defect coverage with the propeller flap, showing primary donor site closure and full-thickness skin grafting. **F** A good result at 12 months postoperatively

**Fig. 4:**
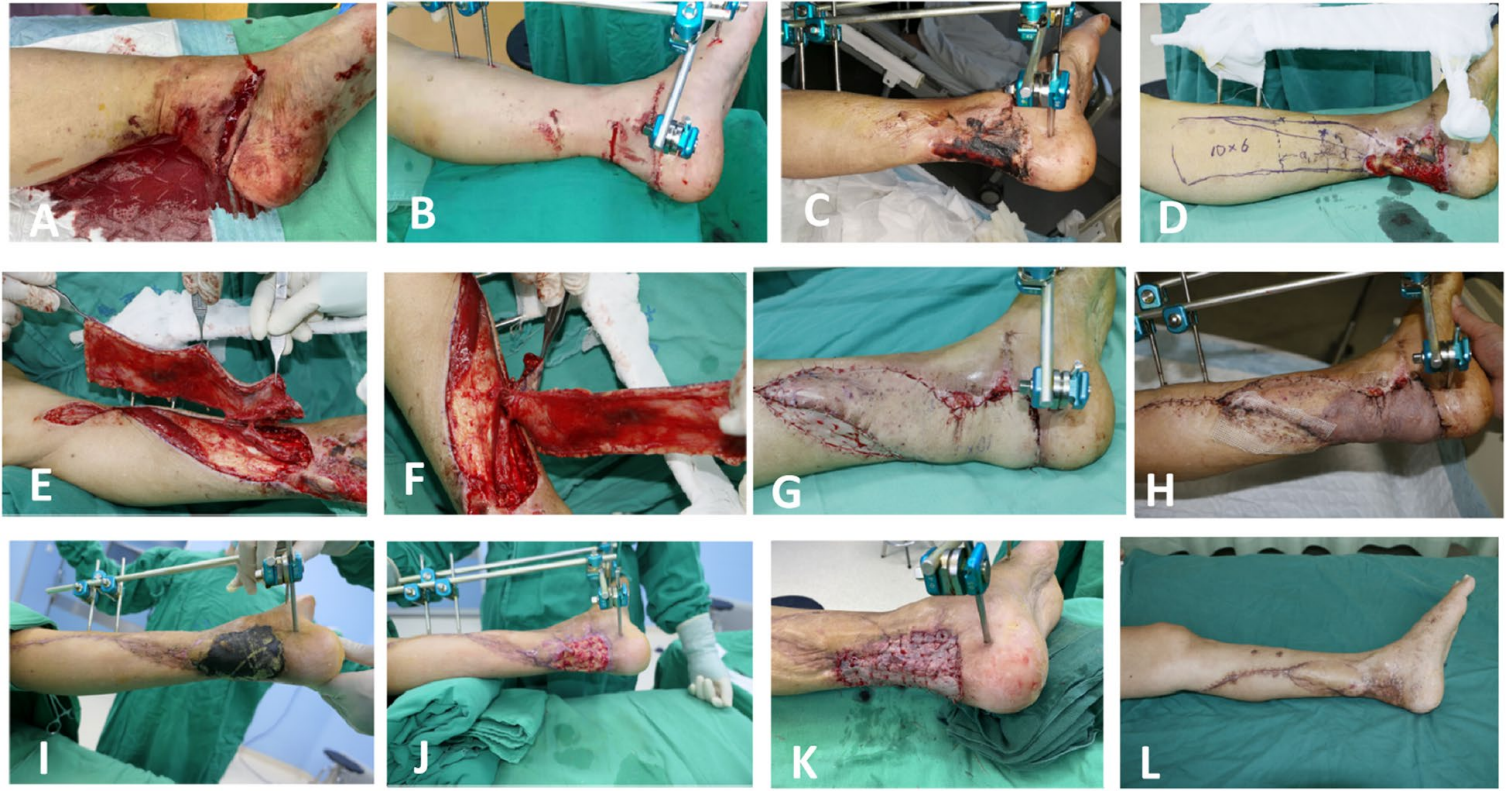
Photographs illustrating the propeller flap operative technique with double pivoting using a posterior tibial artery perforator in a 58-year-old man. **A**, **B**, **C** Post-debridement of the necrosis of the distal third of the posterior leg. **D** Defect with standard marking and perforator location. **E**, **F**, **G** Insetting the flap into the leg defect using the pedicled propeller flap technique. **H**, **I** Partial flap necrosis at 3 weeks postoperatively. **J**, **K** Split-thickness skin grafting performed after debridement. **L** A good result at 14 months postoperatively

(1) The large propeller of the proximally designed propeller flap was cut and rotated to cover the soft tissue defect of the recipient area; the skin from the distal end of the perforator to the wound surface of the recipient area was designed as a spiral. The small paddle covered part of the donor site wound after rotation (Fig. 1). (2) The size of the propeller flap consisted of the size of the wound and the position of the perforator. The length of the flap was equal to the sum of the large oar and the small oar. The length of the large oar was slightly larger than the sum of the length of the long axis of the wound and the length of the small oar. The flap width was slightly larger than the wound width; the length and aspect ratio of the large oar and the small oar were the same as for the originally described pedicled perforator flap. The pedicle perforator flap was used as a reference and did not exceed the cutting range. (3) The axis of the flap conformed to the previous island flap or neurocutaneous nutrition. (4) The shape of the flap was reversed at the end of the oar in accordance with the shape of the wound (Figs. 2, 3, 4).

#### Intraoperative steps

Based on the preoperative identification of the perforator positions and the flap design, the skin was incised along one side of the flap, and separated on the surface or deep surface of the fascia. All perforators were initially saved, and the best perforator was then chosen as the vascular pedicle. The length and position of the flap were adjusted in accordance with the actual position of the perforator. The other side of the flap was cut and the perforator was separated. After thorough hemostasis, the flap was rotated, and the vascular pedicle was checked to ensure that there was no compression, twisting, stretching, or bleeding. The flap was sutured, drainage was placed, and local bracing was performed.

#### Postoperative treatment

After the operation, appropriate antibiotics were selected based on the bacterial culture and drug sensitivity results. If the bacterial culture was negative, empirical broad-spectrum antibiotics were administered. An intramuscular injection of papaverine (30 mg) was routinely administered every 8 h to prevent vasospasm; patients also received adequate fluid replacement, and monitoring of the central vein pressure. Prophylactic anticoagulation therapy was performed for 3–5 days postoperatively. The swelling, color, elasticity, and capillary reaction of the skin flap were closely observed.

#### Complication management

If there was venous stasis (shown as swelling of the skin flap, purple color, and an accelerated capillary reaction), we first determined whether the skin sutures of the vascular pedicle were too tight and there was too much local tension. If necessary, some or all of the vascular pedicle sutures were removed. If suture removal did not improve the venous stasis, blood dripping therapy was implemented immediately; two to three 5-mm incisions were made on the edge of the skin flap, and heparin solution (25 U/ml) diluted with normal saline was administered to maintain incisional bleeding. Patients were closely observed to ensure that blood dripping therapy did not cause excessive blood loss. If skin flap necrosis occurred, the wound was debrided to remove the necrotic tissue, and the activity of the deep fascia of the skin flap was assessed. If the deep fascia was viable, vacuum sealing drainage dressing was applied to the wound, and full-thickness or split-thickness skin grafting was performed in second-stage surgery; if the fascia was necrotic, the fascia was removed, vacuum sealing drainage dressing was applied, and the wound was covered with a flap in second-stage surgery. If necrosis occurred after the purse-pack was removed by skin grafting in the donor area, or the wound of the donor area was directly sutured and the cut edge was necrotic, the necrotic tissue was removed and the deep soft tissue bed was scraped with a curette until the appearance of freshly oozing blood. External dressings were applied and changed until the wound healed.

### Statistical analysis

Statistical analyses were performed using SPSS 23.0 software. Measurement data are expressed as mean ± standard deviation, and compared using the Student’s t-test or the Mann–Whitney U test; count data were compared using the χ^2^ test or Fisher’s exact test. Significant risk factors (*p* < 0.05) in the univariate analyses were included in the multivariable logistic regression analysis. Multivariate logistic regression was used to analyze the factors influencing the occurrence of partial flap necrosis. *P* < 0.05 was considered statistically significant.

## Results

### Clinical results

All 82 patients were followed up after surgery. The mean follow-up time was 12.5 ± 4.2 months (range 3 to 36 months). The flaps completely survived and the wounds healed well in 65 of 82 patients (79.3%); there were 11 cases of partial necrosis (13.4%), and six of complete necrosis (7.3%). Venous crisis occurred in 19 of 82 patients (23.2%); active treatment rescued the flaps in four patients, while the flaps developed necrosis in 15 patients. Arterial crisis occurred in two cases, comprising one case of flap necrosis, and one case in which the flaps were successfully rescued. Deep infection developed in one patient (1.2%) who subsequently developed complete skin flap necrosis. Superficial infection developed in 10 patients (12.3%). Using the posterior tibial artery perforator fascia pedicle flap alone or in combination with simple measures, including dressing changes, secondary suturing, and skin grafting, 91.53% of the wounds were repaired. The donor site healed in the first stage postoperatively, and there were no complications such as ulcers, skin graft necrosis, bone scars, and joint contractures. The results are shown in Table 1, and typical cases are shown in Figs. 2, 3, 4.

Comparison of the flap survival group and necrosis group (including partial and total necrosis).

There were 65 patients in the flap survival group and 17 in the necrosis group. The comparison of the two groups is shown in Table 2. There were no significant differences between the two groups in age, sex, body mass index smoking, prevalence of diabetes, and soft tissue defects (*P* > 0.05). There were no significant differences between the two groups in the length and width of the fascial pedicle, length and width of the flap, and aspect ratio of the flap (*P* > 0.05). The distance between the flap perforator and the wound center did not significantly differ between groups. However, the two groups significantly differed regarding the shortest distance between the skin flap perforator and the wound edge (*P* < 0.05), and the rotation angle of the flap (*P* < 0.05).

### Multivariate analysis

Table 3 shows the results of the multivariate analysis using the dichotomous variable of whether partial necrosis of the skin flap occurred as the dependent variable, and the other variables as independent variables. The shortest distance between the flap perforator and the wound edge was a risk factor for partial necrosis of the flap (*P* = 0.000; OR = 0.806).

**Table 3:** Multiple logistic regression analysis of the risk factors for propeller flap necrosis

Variables	Odds-ratio	95% CI	*P*-value
Nearest distance from perforator to defect location	0.806	0.854–0.952	0.000
Arc of rotation	2.829	1.284–6.670	0.016

### ROC curve analysis

**Fig. 5:**
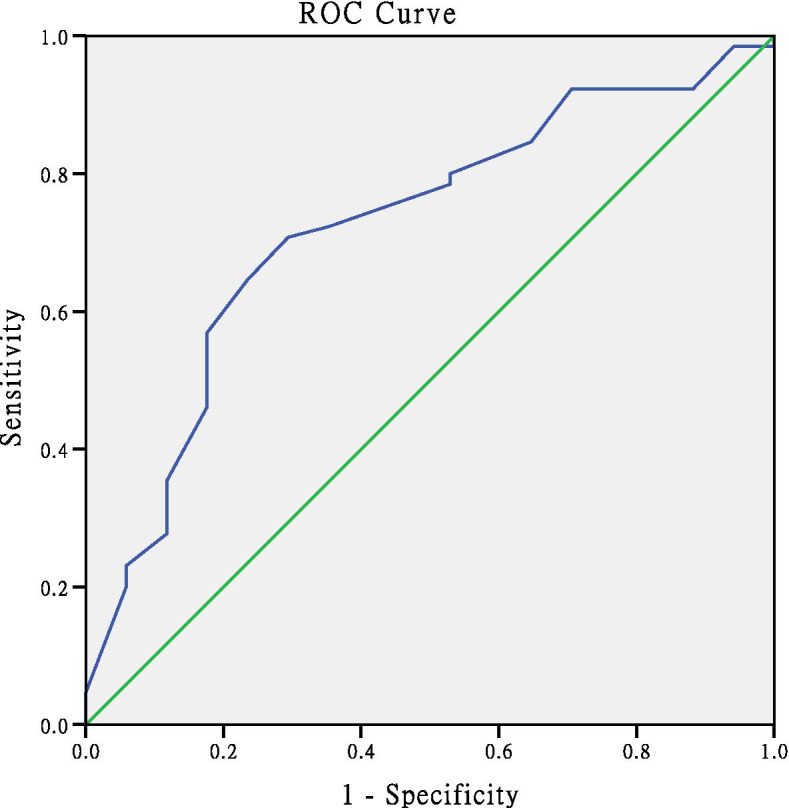
Receiver operating characteristic curves. The optimal cutoff value of the shortest distance from the perforator to the defect is shown for the prediction of surgical outcome

ROC curve analysis showed that when the shortest distance between the perforating branch of the skin flap and the wound surface was less than 3.5 cm, the necrosis rate of the skin flap was significantly increased. The results are shown in Table 4 and Fig. 5.

**Table 4:** Sensitivity, specificity, AUC, and cutoff values of the predictors for propeller flap necrosis

Variable	Sensitivity	Specificity	AUC^a^	Cutoff	*P*-value
Nearest distance from perforator to defect location	69.7%	82.4%	76.1%	3.50	0.005

^a^Area under the curve

## Discussion

A perforator flap is a type of skin flap or subcutaneous flap that is supplied by one (or more) perforator blood vessels that branch from a deeper blood vessel. The isolated perforator is moved and freely dissected together with the overlying tissue, enabling the flap to be moved. Simple translation or transposition of the flap is sufficient in some cases, but when the flap needs to be rotated by more than 90°, it is usually deployed in the manner of a propeller with the perforator used as the axis of rotation, which is called a propeller flap [16].

The propeller flap is a special form of the perforator flap. The advantages of the propeller flap are as follows: (1) because the donor site is located near the defect, the flap is composed of tissue that is similar to the tissue of the recipient site; (2) the donor site arteries and muscles are used to completely or partially close the donor site defect and reduce the morbidity rate of the donor site; (3) lower technical requirements and faster transfer than free tissue flap reconstruction [17–21]. The propeller flap is gaining popularity as a reliable technique for repairing soft tissue defects in the distal leg and ankle; the postoperative appearance is satisfactory, and the procedure is simple, easy to master, and requires a short operation time [8, 22]. The main source arteries for the propeller flap in the distal leg are the posterior tibial artery, anterior tibial artery, and peroneal artery [8].

Although the propeller flap is an established surgical technique, serious complications may still occur and cause failure if not adequately addressed. As the propeller flap is a type of perforator flap, the occurrence of venous return disorder cannot be completely avoided. Venous return disorder is the most common complication of propeller flaps, and is one of the main causes of flap necrosis. Necrosis mainly occurs at the distal end of the flap, but may lead to necrosis of the entire flap in severe cases. Flap necrosis reportedly occurs in 10.77% to 24.00% of cases [23–26]. In the present study, the rate of partial flap necrosis was 20.7%. Flap necrosis may be more likely to occur when the flap perforator is located in the injured area, but this hypothesis lacks objective evidence. There are many reasons for skin flap necrosis. It is currently believed that propeller flap necrosis is influenced by the flap size, pedicle length, and angle of rotation [16, 27].

The first factor causing propeller flap necrosis is the flap size. The size of the propeller flap, especially the size of the large paddle, has a large effect on venous return. After the flap is rotated, if the length of the large paddle is insufficient, the flap is stretched so that it barely covers the wound and causes excessive tension of the vascular pedicle. In addition, flap rotation and other factors increase the risk of venous return disorder. Adequate preoperative preparation and flap design effectively reduce this problem. In the present study, preoperative Doppler examination was performed to determine the locations of the perforating vessels in the distal leg. To identify possible problems and create appropriate strategies, the status of the recipient vessels must be determined before the reconstruction procedure begins (Fig. 3C), especially in complicated cases. When we noticed that it was difficult to differentiate a perforator from the main vessel, we preoperatively performed 64-slice CTA (GE Optima CT660, Yokohama, Japan) using a General Electric Light speed VCT Scanner preoperatively. Over the past 30 years, CTA has emerged as an alternative noninvasive modality with many clinical applications. For example, Demirtas et al. showed that the radiographic and operative findings regarding the availability of the recipient vessels for anastomosis were correlated in 21 of 23 patients [28]. Numerous studies have confirmed the value of CTA for assessing vascular regions in the cranium, head and neck, thorax, and abdomen [29, 30].

The rotation point of the perforator propeller flap depends on the position of the perforator fulcrum. In theory, the closer the perforator fulcrum is to the wound, the greater the length of the flap that can be cut. The size of the skin flap should be designed in accordance with the size of the wound, and the position and diameter of the perforating vessels. The large paddle is located near the axis of rotation, and its length should be 0.5 to 1.0 cm longer than the distance from the point of rotation to the most distal end of the wound. The small paddle is located between the rotation point and the wound surface. The width of the flap should be 0.5–1.0 cm wider than the wound surface, and is determined by the thickness of the subcutaneous fat. However, the closer the perforator is to the wound, the greater the impact on the wound. Wound inflammation reportedly damages the perforator blood vessels and causes necrosis [31]. Our study found that the necrosis rate was significantly higher when the perforator was farther away from the flap. Furthermore, ROC curve analysis showed that the flap necrosis rate increased when the distance from the perforator to the wound was less than 3.5 cm (sensitivity 69.7%; specificity 82.4%) (Table 4). We consider that there were three reasons for the increased risk of flap necrosis in cases where the perforator was closer to the flap. 1. The degree of inflammation. The inflammatory response of the flap is related to the injury mechanism/wound contamination severity. In the present study, the injuries were caused by high-energy trauma, excluding the patients with infections caused by soil and sewage. 2. The shape of the skin wound. The present study assessed the distance from the perforator to the center of the flap, while the distance from the perforator to the wound center did not significantly affect the flap necrosis rate (Figs. 2B, 3B,  4D). 3. The time from injury to wound coverage, and the inflammatory reaction period. As inflammation peaks at 7–12 days after injury, it is optimal to cover the wound before or after this period. The exposed wound needs to be treated with standard dressing changes.

Previous studies have also shown that the flap width affects the survival of the flap, as the anastomoses between the perforators of the main blood vessels in the calf are almost all choke anastomoses [32]. Therefore, the wider the flap, the farther the edge of the flap will be from the axis of rotation, and the greater the decrease in the diameter of the vascular network and the pressure of the blood flow; furthermore, due to the special anatomical structure of the lower leg, when the flap position is lower, the wider edge of the flap approaches or even surpasses the midline of the front and rear of the calf, which directly leads to partial necrosis of the flap.

The second factor affecting propeller flap necrosis is the length of the vascular pedicle. After the flap is rotated, the pedicle must be kept under appropriate tension. A vascular pedicle that is too short causes excessive local tension due to traction, which causes venous return disorder. Intraoperatively, the pedicle vessel should be dissected in the direction of the source vessel as much as possible to create a length of at least 3 cm and a width of at least 1 mm [33]; this significantly reduces the risk of blood vessel deformation after rotation. The caliber of the blood vessel must also be considered. Preoperative Doppler examination must be performed to locate the perforator position and select a perforator with a suitable caliber as the direct nutrient vessel for the flap [34].

The third factor affecting propeller flap necrosis is the flap rotation angle. The propeller flap needs to rotate at a large angle of up to 180°. The perforating vessels, especially the perforating veins, are easily compressed by the surrounding deep fascia fiber bundles due to their thin wall and low pressure [35]. Therefore, the vascular pedicle usually needs to be naked, and different rotation directions (clockwise or counterclockwise) should be assessed intraoperatively; the rotation direction that causes the smallest twist of the pedicle should be selected [36]. The complication rate of propeller flap reconstruction is higher in the extremities than in the trunk. This is because the trunk has relatively abundant perforators and large perforator areas connected by blood vessels, which may aid in the safe harvest of flaps, thereby reducing the incidence of complications [11, 26, 37–40].

Finally, previous studies suggest that other risk factors for flap necrosis include patient age over 50 years, smoking history, and diabetes mellitus [3, 27], but these factors did not significantly affect the development of flap necrosis in the present study.

## Limitations

This study has some limitations. First, the sample size was relatively small, so a significant relationship between potential risk factors and complications may be hidden. Second, this was not a prospective study. Third, we did not perform this kind of surgery on a series of patients with bone defects in other parts, which means that there may be selective bias in choosing this surgical technique. Fourth, there was no control group who underwent other surgical techniques, such as free flap reconstruction. However, although we did not compare the results of perforator and free flap reconstructions, a recent meta-analysis study reported that the overall failure and complication rates are similar for free flaps (19.0%) and perforator flaps (21.4%) [41]. Despite these study limitations, to the best of our knowledge, this is the first quantitative and accurate study of the relationship between the position of the propeller flap and the wound surface. Therefore, our data may be considered useful pilot data for future multicenter studies on this topic. This research will help surgeons identify potential risk factors and choose the appropriate surgical method to repair soft tissue defects of the lower limbs.

## Conclusions

The propeller flap has been widely used in reconstruction of defects on the trunk and limbs. The present study showed that when the distance between the flap perforator and the wound surface is less than 3.5 cm, the necrosis rate of the flap is significantly increased; that is, the effective safe distance is 3.5 cm. When the perforator is within this distance, the propeller skin needs to be selected more carefully.

## Data Availability

The datasets used and/or analyzed during the current study are available from the corresponding author on reasonable request.

## Abbreviations

PPF: Perforator Propeller Flap
ROC: In receiver operating characteristic
CTA: Computed tomographic angiography
